# Debottlenecking mevalonate pathway for antimalarial drug precursor amorphadiene biosynthesis in *Yarrowia lipolytica*

**DOI:** 10.1016/j.mec.2019.e00121

**Published:** 2020-01-02

**Authors:** Monireh Marsafari, Peng Xu

**Affiliations:** Department of Chemical, Biochemical and Environmental Engineering, University of Maryland Baltimore County, Baltimore, MD, 21250, USA

**Keywords:** Amorphadiene, Antimalarial, Heterologous expression, Mevalonate pathway, Metabolic engineering, *Yarrowia lipolytica*

## Abstract

World Health Organization reports that half of the population in developing countries are at risk of malaria infection. Artemisinin, the most potent anti-malaria drug, is a sesquiterpene endoperoxide extracted from the plant *Artemisia annua*. Due to scalability and economics issues, plant extraction or chemical synthesis could not provide a sustainable route for large-scale manufacturing of artemisinin. The price of artemisinin has been fluctuating from 200$/Kg to 1100$/Kg, due to geopolitical and climate factors. Microbial fermentation was considered as a promising method to stabilize the artemisinin supply chain. *Yarrowia lipolytica,* is an oleaginous yeast with proven capacity to produce large quantity of lipids and oleochemicals. In this report, the lipogenic acetyl-CoA pathways and the endogenous mevalonate pathway of *Y. lipolytica* were harnessed for amorphadiene production. Gene overexpression indicate that HMG-CoA and acetyl-CoA supply are two limiting bottlenecks for amorphadiene production. We have identified the optimal HMG-CoA reductase and determined the optimal gene copy number for the precursor pathways. Amorphadiene production was improved further by either inhibiting fatty acids synthase or activating the fatty acid degradation pathway. With co-expression of mevalonate kinase (encoded by Erg12), a push-and-pull strategy enabled the engineered strain to produce 171.5 ​mg/L of amorphadiene in shake flasks. These results demonstrate that balancing carbon flux and manipulation of precursor competing pathways are key factors to improve amorphadiene biosynthesis in oleaginous yeast; and *Y. lipolytica* is a promising microbial host to expand nature’s biosynthetic capacity, allowing us to quickly access antimalarial drug precursors.

## Highlights

• Endogenous acetyl-CoA and mevalonate pathway were harnessed for amorphadiene synthesis.
• Expression of native untruncated HMG-CoA reductase (HMG1) removes rate-limiting steps.
• Balancing ADS, HMG1 and MVK activity effectively pull FPP flux toward amorphadiene.
• Activation of fatty acid degradation pushes carbon flux toward HMG-CoA pathways.
• A push-and-pull strategy boosts amorphadiene production to 171.5 ​mg/L in shake flasks.

## 1 Introduction

Nearly half of the world’s population in developing countries are at risk of malaria infection. In 2017, there were roughly 219 million malaria cases in 87 countries and an estimated 435,000 malaria deaths. African regions carry a disproportionately high share of the global malaria crisis. The best available treatment, particularly for *Plasmodium falciparum* malaria, is artemisinin-based combination therapy (ACT) (2019). Artemisinin is a sesquiterpene lactone with an unusual endoperoxide structure, and so far the plant *Artemisia annua* is the only commercially feasible source of artemisinin for drug formulations. Numerous derivatives of artemisinin have also been synthesized and tested against malaria parasites. Artemisinin specifically and selectively inhibits the sarco/endoplasmic reticulum Ca ATPase (SERCA) of *P. falciparum* after activation by iron ions. Artemisinin has also been proven as an anti-cancer natural product against breast and colon cancer and leukemia (Weathers et al., 2006; Wen and Yu, 2011). Due to safety and economic issues, traditional plant extraction or chemical synthesis could not provide a scalable route for large-scale manufacturing of artemisinin. Therefore, it is necessary to seek alternative sources which are economically viable for the large-scale commercial production (Weathers et al., 2006).

**Fig. 1:**
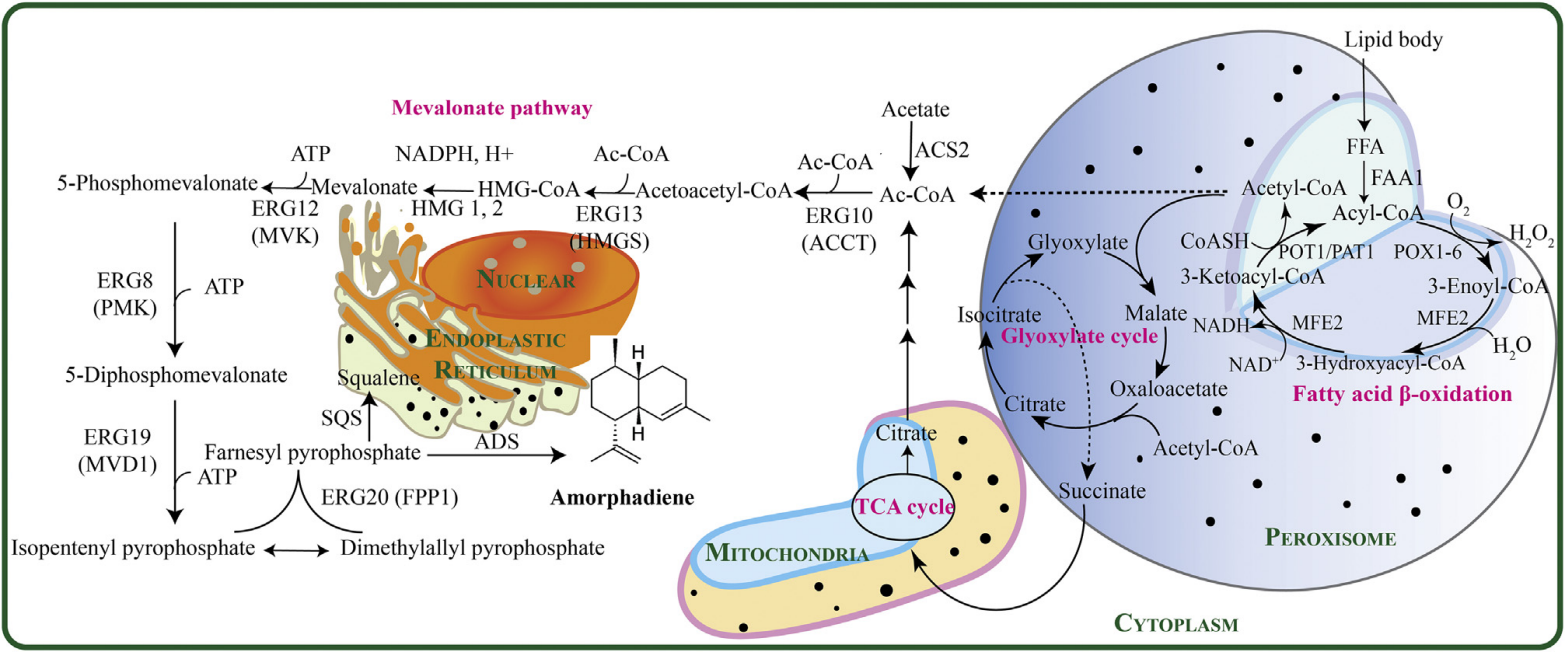
Biosynthetic pathway of amorphadiene production in *Y. lipolytica.* In the MVA pathway, acetoacetyl-CoA thiolase (ACCT), 3-hydroxyl-3-methyglutaryl CoA synthase (HMGS), 3-hydroxyl-3-methyglutaryl CoA reductase (HMG1), mevalonate kinase (MVK), mevalonate-5-phosphate kinase (PMK), mevalonate pyrophosphate decarboxylase (MVD1) and IPP isomerase (IPI) all play important roles. Gene, enzyme and metabolite symbols: FFA, free fatty acids; ACS2, acetyl-coA synthetase; POX1-6, fatty acyl-CoA oxidases; MFE2, multifunctional enzyme type 2; POT1, 3-ketoacyl-CoA thiolase; ERG10 (ACCT and PAT1), Acetyl-CoA C-acetyltransferase; ERG13 (HMGS), 3-hydroxy-3-methylglutaryl-CoA synthase; HMG 1, 2, HMG-CoA reductase; ERG12 (MVK), Mevalonate kinase; ERG8 (PMK), Phosphomevalonate kinase; ERG19 (MVD1), Mevalonate pyrophosphate decarboxylase; ERG20 (FPP1), Farnesyl pyrophosphate synthetase; SQS, Squalene synthase; AMD, Amorphadiene synthase.

Amorphadiene is the direct sesquiterpene olefin precursor to artemisinin, derived from the native farnesyl pyrophosphate precursors by the action of amorphadiene synthase (ADS) (Fig. 1**)** (Bouwmeester et al., 1999). Isopentenyl diphosphate (IPP) and dimethylallyl diphosphate (DMAPP) are two universal isoprenoids precursors, which are biosynthesized via mevalonate originating from acetyl-CoA. Plants primarily use mevalonate (MVA) pathway for the biosynthesis of isoprenoids. Most of yeast share the MVA pathway with plants with proven activity to convert acetyl-CoA to IPP, which is subsequently isomerized to DMAPP (Martin et al., 2003). The formation of FPP is catalyzed by farnesyl pyrophosphate synthase encoded by Erg20 (Fig. 1) (Wen and Yu, 2011).

Metabolic engineering and synthetic biology allow biochemical engineers to translate heterologous pathways from natural plant to an appropriate microbial host (Xu et al., 2013)*.* With innate mevalonate pathway (MVA), yeast strains are robust hosts for metabolic engineering and industrial production of isoprenoids derivatives (Krivoruchko and Nielsen, 2015). With ease of extraction and purification as well as cost effectiveness, engineered yeast produce 2- to 3-fold amount of artemisinic acid biomass within 4–5 days compared to *A. annua* plant suspension culture in several months (Ro et al., 2006; Zeng et al., 2008; Baadhe et al., 2013). Synthetic biology efforts for the production of amorphadine were first carried out in *E. coli* in 2003. The rate-limiting enzymes of the native DXP pathway and the genes encoding isoprenoid synthesis were overexpressed to produce the antimalarial drug precursor amorphadiene (Martin et al., 2003). Introducing *S. cerevisiae* heterologous mevalonate pathway into *E. coli* along with optimizing nitrogen source in the fermentation process improved amorphadiene production up to 25 ​g/L (Tsuruta et al., 2009). Overexpression of all enzymes of the mevalonate pathway including ERG20 in *S. cerevisiae* led to produce 40 ​g/L amorphadiene, which is the highest amount ever reported. However, the amount of artemisinic acid was not improved in parallel with amorphedine (Westfall et al., 2012). While efforts to maximize the production of antimalarial drugs continued in *S. cerevisiae*, feeding a mixed carbon sources containing glucose and ethanol improves amorphadiene production to 12 ​g/L in wild type strain (Paddon et al., 2013). Recently, engineering of transcription, translation and enzyme for production of viridiflorol in *E. coli* leads to produce 30 ​g/L amorphadiene (Shukal et al., 2019). Amyris optimized the mevalonate pathway in yeast to produce very high-level of amorphadiene and artemisinic acid in ethanol-feeding bioreactors with isopropyl myristate (IPM) as overlay (Westfall et al., 2012; Paddon et al., 2013; Paddon and Keasling, 2014). However, this process could not meet the increasing demand for artemisinin-based combination therapy with cost that is comparable to the agriculture-sourced artemisinin (Peplow, 2016). This is necessary to seek alternative biological pathways and chassis hosts to further lower the artemisinin-manufacturing cost and stabilize the volatile artemisinin market price*.*

*Yarrowia lipolytica* is a dimorphic, non-pathogenic oleaginous yeast (Zinjarde et al., 2014), which has been world-wide attracted with its high secretion capacity, strong acetyl-CoA and malonyl-CoA flux (Liu et al., 2019a, Liu et al., 2019b; Lv et al., 2019b, Lv et al., 2019a), large collection of genetic tools (Wong et al., 2017; Lv et al., 2019b, Lv et al., 2019a; Yang et al., 2020), and the “generally regarded as safe” (GRAS) status (Liu et al., 2013; Liu et al., 2019a, Liu et al., 2019b). It is a superior platform for cost-efficient production of biochemicals or oleochemicals derived from fatty acids, lipids and acetyl-CoAs (Zhu and Jackson, 2015; Qiao et al., 2017; Xu et al., 2017). Recent studies demonstrate that *Y. lipolytica* can grow on a wide range of cheap renewable feedstocks (Ledesma-Amaro and Nicaud, 2016) such as glucose, fructose, glycerol and hydrophobic substrates including volatile fatty acids and alkanes (Workman et al., 2013). In addition, the endogenous mevalonate pathway of *Y. lipolytica* has been harnessed to produce large amount of carotenoids recently (Gao et al., 2017), indicating its capacity to accommodate high HMG-CoA flux.

With the introduction of codon-optimized *A. annua-*derived amorphadiene synthase (ADS), a trace amount of amorphadine (~23.8 ​mg/L) could be rapidly detected. To debottleneck amorphadiene biosynthesis, we firstly investigated the possible rate-limiting steps and assessed a panel of yeast-derived 3-hydroxyl-3-methyglutaryl CoA reductase (HMG-CoA reductase) in *Y. lipolytica*. We then evaluated the catalytic performance of numerous mevalonate precursor enzymes, including 3-hydroxyl-3-methyglutaryl CoA synthase, mevalonate kinase, phosphomevalonate kinase, mevalonate diphosphate decarboxylase and farnesyl pyrophosphate. We identified that the formation of acetoacetyl-CoA and mevalonate are early rate-limiting steps. These genes were coexpressed with the endogenous HMG1 to improve amorphadiene production, along with the inhibition of fatty acids synthase. Comprehensive investigation of biosynthetic pathway indicates that acetyl-CoA supply is a late rate-limiting step for amorphadiene production, this bottleneck was removed by activating the fatty acids degradation pathways. The engineered strain produces 171.5 ​mg/L of amorphadiene in shake flasks, a seven-fold improvement compared to the parental strain. This work validated that *Y. lipolytica* is an excellent yeast platform for production of plant-derived anti-malarial drug producers from simple feedstocks.

## 2 Results and discussion

### 2.1 Reconstruction and optimization of amorphadiene biosynthesis in *Y. lipolytica*

HMG1, a rate-limiting step of the mevalonate pathway, catalyzes the reduction of HMG-CoA to mevalonate and is the key enzyme in the mevalonate pathway (Goldstein and Brown, 1990). The activity of the native HMG1 is insufficient to balance flux in the engineered pathway. Modulation of HMG1 expression will eliminate the pathway bottleneck and increase mevalonate flux and improve the amorphadiene titer (Pitera et al., 2007).

**Fig. 2:**
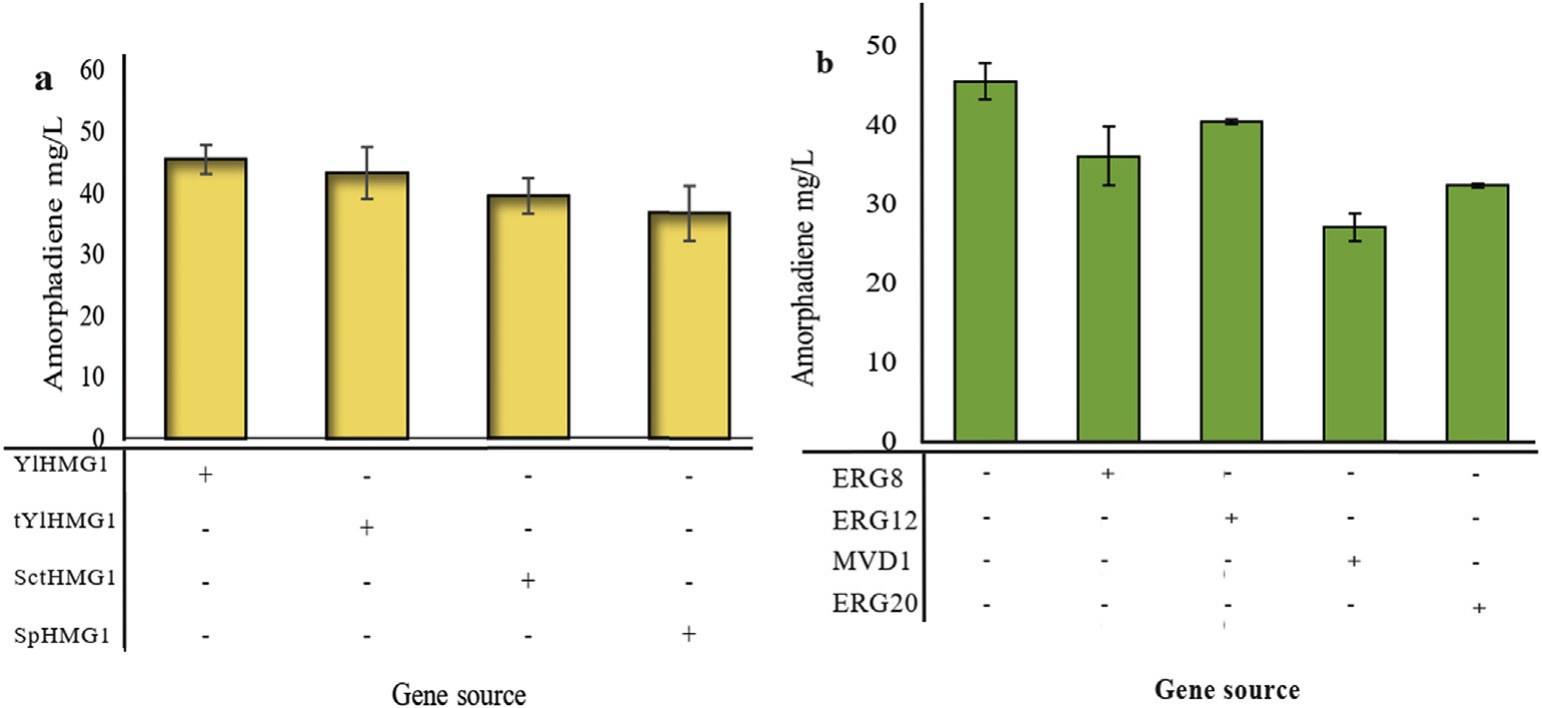
Screening of rate-limiting steps in native mevalonate pathway to improve amorphadiene production. **a.** Screening of HMG1 gene from different origin. **b.** Identifying of possible rate-limiting steps to improve amorphadiene production. All genes were combined with AaADS-ylHMG1.

By simply introducing the codon-optimized amorphadien synthase (ADS), the strain with ADS produced about 23.8 ​mg/L of amorphadiene, analyzed by GC-FID with beta-caryophyllene as internal standard. Harnessing the native HMG-CoA pathway, we then reconstructed and validated metabolic pathway with HMG1 genes from different sources (SpHMG1, ScHMG1, YlHMG1and tYlHMG1) in the *Y. lipolytica* Po1g host. We observed that all constructs containing ADS and HMG1 led in the production of amorphadiene, ranging from 36.67 ​mg/L to 45.42 ​mg/L (Fig. 2a). It is noteworthy, the two top strains (Fig. 2a) contain the same source of HMG1 gene (the original and truncated form) from *Y. lipolytica*, indicating the native gene is sufficient to support the mevalonate flux that is required for amorphadiene synthesis. We next investigated the effect of oleic acid supplementation on cell growth and amorphadiene accumulation. The results showed that supplementation of oleic acid may negatively impact mevalonate pathway in *Y. lipolytica* and decrease the amount of amorphadiene accumulation to 32.5 ​mg/l in the shake flasks (Supplementary Fig. S1). Previous investigations confirmed supplementation of oleic acid in the medium led to lipid accumulation in Y. lipolytica and the lipid increase is dramatically influenced by the fatty acid composition of the culture medium (Athenstaedt et al., 2006; Beopoulos et al., 2008).

To obtain the suitable precursor supply and achieve high amorphadiene titer, we introduced ERG12, ERG8, MVD1 and ERG20 into our engineered strain. We found that strain Po1g containing both HMG1 (derived from *Y. lipolytica*) and ERG12 led to the highest amorphadiene production around 40 ​mg/L. However, this titer was not significantly different from the control sample (Fig. 2b). Mevalonate kinase catalyzes the conversion of mevalonic acid into mevalonate-5-phosphate, a key pathway intermediate, using ATP as cofactor. We speculated that the initial reaction of mevalonate to phosphomevalonate by ERG12 is one of the rate-limiting steps in the amorphadiene production. The expression of ERG12 is tightly regulated, at transcriptional level in a similar manner of HMG1. Geranylgeranyl pyrophosphate and farnesyl pyrophosphate are two of the most important precursors that feedback inhibit ERG12, demonstrating that non-sterol isoprenoid could have a key role in regulation of ERG12 (Hinson et al., 1997; Murphy et al., 2007).

### 2.2 Increasing gene copy number to improve amorphadiene titer

**Fig. 3:**
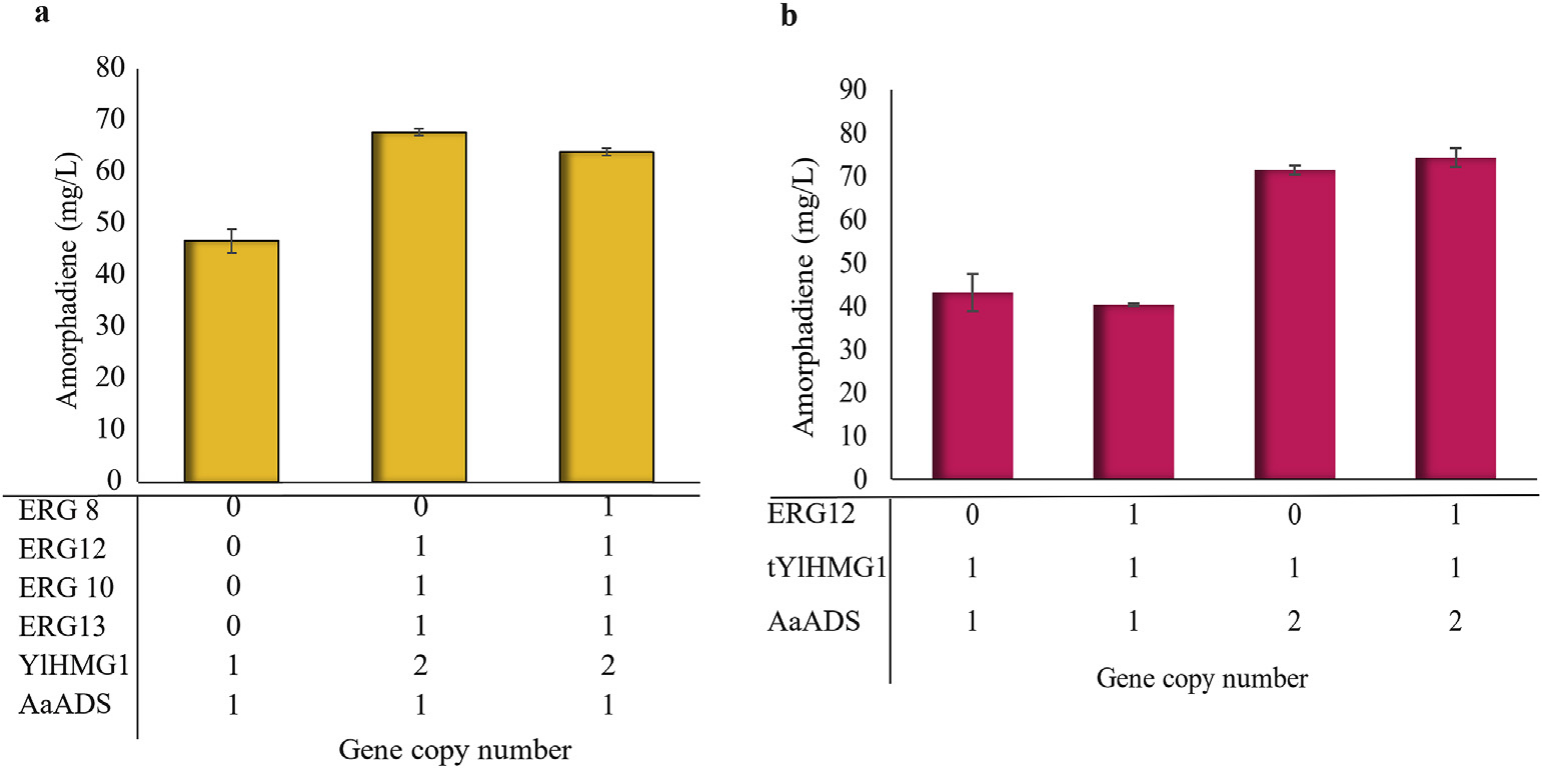
Overcoming rate-limiting steps by tuning gene copy numbers. **(a)** Optimizing mevalonate pathway and YlHMG1 gene copy number to improve amorphadiene production. **(b)** Rate-limiting step analysis by tuning AaADS gene copy number.

Gene copy numbers are an important factor determining gene expression levels. For some burdensome or toxic genes, medium or single copy gene numbers are the only way to avoid formation of inclusion bodies or the accumulation of toxic intermediates (Nadler et al., 2018). Considering that the conversion of acetyl-CoA to mevalonate is the only metabolic route to amorphadiene production, expression of ERG10, HMG1 and ERG13 may be imbalanced and limits the pathway efficiency. Previous studies have proven the benefits to balance gene expressions on these metabolic nodes (Paddon and Keasling, 2014). We next sought to balance the gene expression in mevalonate pathway, by overexpressing ERG10, HMG1 and ERG13 along with ERG12 and ERG8 (Po1g/AYlH1_x2_E13E10E12 and Po1g/AYlH1_x2_E13E10E12E8 strains). Amorphadiene was increased by more than 1.40-fold compare to control (Po1g/AYlH) when the gene copy number of YlHMG1 was increased from one to two along with other upstream genes (Fig. 3a). While the upstream mevalonate pathway is an important factor for amorphadiene production, the ADS copy number has also been suggested as a rate-limiting factor (Anthony et al., 2009). For this reason, we increased the copy number of ADS gene with our YaliBrick assembly platform (Wong et al., 2017). With two copies of ADS, the engineered strain (Po1g/A_x2_tYlH strain) produced 71.39 ​mg/L amorphadiene from glucose, a 1.65-fold increase compared to the control cassette (Po1g/AtYlH) (Fig. 3b). With the increasing AaADS copy number, the metabolic balance may be shifted to a different equilibrium point. We next focused on removing the rate-limiting step in the mevalonate pathway by adding additional copy of ERG12. It should be noted that we used truncated form of YlHMG1 named tYlHMG, because the larger size of native gene (YlHMG1, ~3 ​kb), contains various restriction enzyme sites limiting our ability for subcloning and gene assembly. Increasing the copy number of ADS along with overexpression of ERG12 (Po1g/A_x2_tYlHE12 strain) did not have significant effect on amorphadiene production compared to Po1g/A_x2_tYlH strain, but amorphadiene production was increased by 1.83-fold compared to the control strain Po1g/AYlHE12 (Fig. 3b), possibly due to the unbalanced gene expression between the upstream and downstream pathway.

### 2.3 Improving amorphadiene titer by inhibiting fatty acids synthesis

**Fig. 4:**
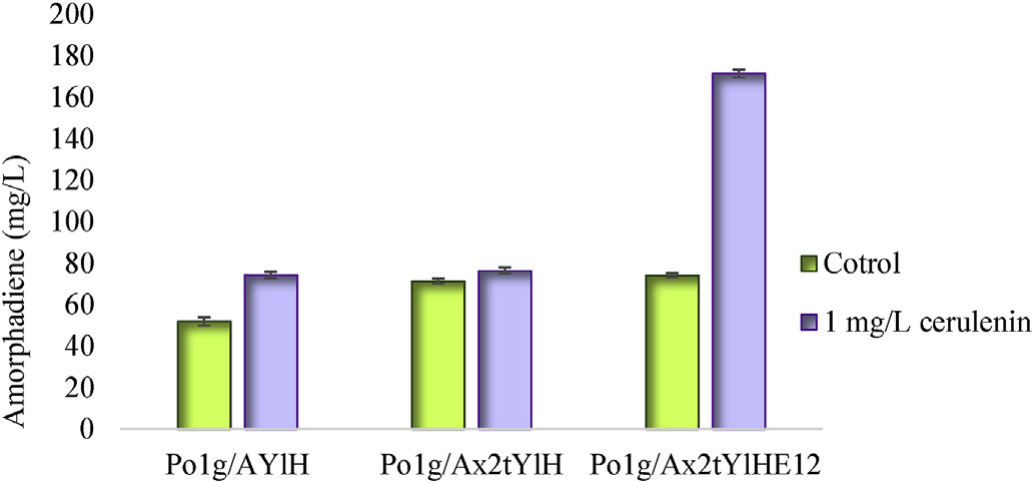
Effect of 1 ​mg/L cerulenin on amorphadiene production carried out for Po1g/AYlH (as a parental strain), Po1g/A_x2_tYlH and Po1g/A_x2_tYlHE12 strains compared to the same strains without supplementation of cerulenin (control).

Acetyl-CoA is the central code in the mevalonate pathway, which is primarily used as precursor for fatty acids and lipid synthesis. Therefore modulating acetyl-CoA competing pathways in the cytoplasm is crucial to improve heterologous metabolite production (Chen et al., 2013). As illustrated in Fig. 1, acetyl-CoA is formed in the different compartments of cell, such as peroxisome, mitochondria and cytosol, while for *Y. lipolytica*, most of the cytosolic acetyl-CoA is directed toward lipid biosynthesis. Supplementation of cerulenin will inhibit lipid biosynthesis and lead the carbon flux toward amorphadiene biosynthesis (Zha et al., 2009). Cerulenin is an inhibitor that can block lipids synthesis specifically by inhibiting 3-ketoacyl-ACP synthases I and II, which are responsible for loading malonyl-CoA units to the elongating fatty acyl chains (Davis et al., 2000; Markham et al., 2018). We next investigate the effect of cerulenin on amorphadiene production. To find the best cerulenin dosage for treatment *Y. lipolytica* cells, we used 0, 1 and 2 ​mg/L cerulenin (Supplementary Fig. S2). The results showed that using 1 ​mg/L cerulenin in the shake flasks containing strain Po1g/A_x2_tYlHE12, improved amorphadiene titer by 231.13%, reaching 171.455 ​mg/L compared to control (no cerulenin) (Fig. 4). Two other strains are not affected by cerulenin because they lacked ERG12 which was mevalonate kinase that serve as a rate-limiting sink pathway for acetyl-CoA. These results may confirm that a push-and-pull strategy, by simultaneously mitigating acetyl-CoA competing pathways (i.e. with cerulenin to inhibit acetyl-CoA consumption) and enhancing acetyl-CoA-sink pathways (ERG12), might be the most efficient factors to improve amorphadiene production in *Y. lipolytica*.

### 2.4 Boosting amorphadiene production by activating fatty acid degradation pathway

Cellular fatty acid was degradated into acetyl-CoA units through β-oxidation, which in yeast occurs exclusively in peroxisomes. In *Y. lipolytica*, this cyclic β-oxidation reaction is carried out by, in order, acyl-CoA oxidase, multifunctional enzyme type 2 (MFE2) and 3-ketoacyl-CoA thiolase (POT1). POT1 breaks down 3-ketoacyl-CoA to produce acetyl-CoA (Fig. 1). Acetyl-CoA acetyltransferase (PAT1) which encodes acetoacetyl-CoA thiolase is also responsible for providing acetoacetyl-CoA units (Smith et al., 2000), which could be used as a direct precursor to synthesize HMG-CoA. The promising result of using cerulenin to downregulate fatty acids synthesis, encourage us to use POT1 and PAT1 as potential gene targets to improve acetyl-CoA and acetoacetyl-CoA pool and subsequently improve amorphadiene titer.

**Fig. 5:**
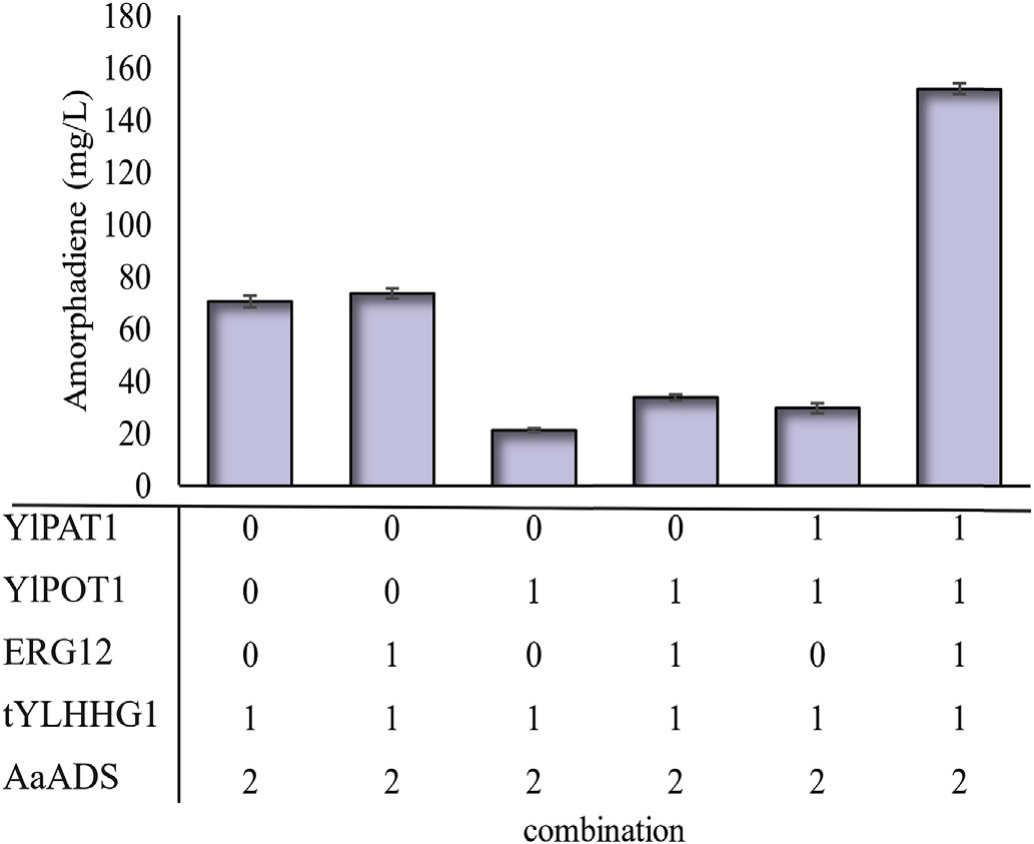
Improving amorphadiene production by activating fatty acids degradation pathway. Identification of possible rate-limiting steps by overexpression of acetyl-CoA C-acetyltransferase (PAT1) and 3-ketoacyl-CoA thiolase (POT1). The related genes were overexpressed in strains Po1g/Ax2tYlH and Po1g/Ax2tYlHE12.

For this aim, we first tested *Y. lipolytica* POT1 gene and introduced these constructs into Po1g/A_x2_tYlH and Po1g/A_x2_tYlHE12 to generate strains Po1g/A_x2_tYlHP and Po1g/A_x2_tYlHE12P, respectively. The resulting strain produced maximum 21.39 ​mg/L amorphadiene which is less than the controls (Fig. 5). This significantly decreased isoprenoid production, indicating that high acetyl-CoA flux may inhibit the key steps in the mevalonate pathway. Subsequently we introduced PAT1 gene into Po1g/A_x2_tYlHP and Po1g/A_x2_tYlHE12P, to convert the accumulated acetyl-CoA to acetoacetyl-CoAs. These results showed the coexpression of PAT1 and POT1 can improve amorphadiene production to 214.95%, reaching 152.185 ​mg/L (Fig. 5). This drastic increase in amorphadiene production with PAT1 and POT1 is likely due to the shift of metabolic rate-limiting steps: expression of PAT1 could effectively remove overflow of acetyl-CoA and relieve the metabolic inhibition of acetyl-CoA on the key steps in mevalonate pathway. These results indicated that acetyl-CoA and mevalonate are two most important rate-limiting steps for heterologous production of amorphadiene in *Y. lipolytica*. Apart from flux balance, we speculate that the supply of acetyl-CoA and NADPH might be the limiting factors at the initial stage. As the cell enters stationary phase, the accumulation of oxidative stress (Xu et al., 2017) may cause electron transport chains lose their proton driving-force, and therefore, energetic ATP supply might be the liming factor (Hollinshead et al., 2014; Varman et al., 2014; Wu et al., 2015). The shift of metabolic bottleneck from acetyl-CoA/NADPH to ATP should be systematically investigated to unlock the potential of *Y. lipolytica* as host for amorphadiene production. Recent literature studies on *E. coli* metabolism (Hollinshead et al., 2014; Varman et al., 2014; Wu et al., 2015) support the hypothesis of the shifting of metabolic bottleneck, which provides the foundation for our future work.

## 3 Conclusions

The optimization of isoprenoid production and balancing of mevalonate pathway in oleaginous yeast has validated that *Y. lipolytica* is a prosperous heterologous host for amorphadiene production. Although microbial cell factories for natural compounds production have been suggested to solve existing problems, optimization of heterologous pathways and host strains are necessary steps to reach maximal titers. *Y. lipolytica* is a well-known oleaginous yeast with an extensive array of genetic toolbox which permits us to perform genetic modifications to produce valuable pharmaceutical and natural products. In this research, we aim to increase the production of amorphadiene, we assembled the genes encoding the mevalonate pathway and amorphadiene synthase into mono-cistronic form and expressed them in *Y. lipolytica* Po1g strains. Our engineering effort led us to capitalize the endogenous mevalonate pathway and the high lipogenic acetyl-CoA flux. This attempt leads us to identify that acetoacetyl-CoA thiolase, mevalonate kinase and HMG-CoA reductases are important rate-limiting steps and causing bottlenecks for amorphadiene production in *Y. lipolytica*. ATP-dependent activation of mevalonate by mevalonate kinase is the most important step to sink HMG-CoA and improve amorphadiene titer. We further identified the optimal gene copy number for ADS and HMG1 to unleash the amorphadiene production potential. Through downregulating precursor competing pathways by supplementation of cerulenin, we demonstrate the acetyl-CoA pool is a crucial factor for improving mevalonate flux toward high amorphadiene titer. We then overexpressed POT1 and PAT1 to activate the beta-oxidation pathway and synergistically improved amorphadien titer. The applied engineering strategies effectively removed pathway bottlenecks and increased amorphadiene titer to seven-fold compared to control strain. The results of this study present a model system for engineering oleaginous *Y. lipolytica* as microbial workhorse for potential production of bioactive molecules of therapeutic and industrial value.

## 4 Material and methods

### 4.1 Genes, plasmids and strains

Genes encoding *A. annua* amorphadiene synthase (AaADS) were optimized and synthesized by invitrogen company (USA). *Y. lipolytica* 3-ketoacyl-CoA thiolase (YlPOT1), *Y. lipolytica* Acetyl-CoA C-acetyltransferase (YlPAT1), *Y. lipolytica* acetoacetyl-CoA thiolase (ERG10), *Y. lipolytica* 3-hydroxy-3-methylglutaryl-CoA synthase (YlHMGS), *Y. lipolytica* 3-hydroxy-3-methylglutaryl-CoA reductase (YlHMG1) and its truncated form (tYlHMG1), *Y. lipolytica* mevalonate kinase (ERG12), *Y. lipolytica* phosphomevalonate kinase (ERG8), *Y. lipolytica* mevalonate diphosphate decarboxylase (YlMVD1) and *Y. lipolytica* farnesyl pyrophosphate (ERG20), were amplified from *Y. lipolytica* Po1g genomic DNA by PCR reaction. *Saccharomyces cerevisiae* 3-hydroxy-3-methylglutaryl-CoA reductase (ScHMG1), and *Streptococcus pneumoniae* 3-hydroxy-3-methylglutaryl-CoA reductase (SpHMG1) (Miller and Kung, 2018) were codon-optimized and synthesized by Genewiz. All the genes included in this paper were listed in Supplementary Table S1.

Plasmid pYLXP’ was previously designed and maintained in our laboratory (Xu et al., 2016). *Escherichia coli* strain NEB5α was used for plasmid construction and maintenance. The strain was grown in LB media (Liquid media or plate containing 15 ​g/L agar) at 37 ͦC supplemented with ampicillin (100 ​mg/ml) for selection. *Y. lipolytica* strain Po1gΔLue was engineered to produce amorphadiene and used as the parent strain for all engineered yeast strains. All the strains included in this research were shown in Supplementary Table S2**.**

### 4.2 Molecular cloning and pathway construction

YlHMGS, YlHMG1, tYlHMG1, ScHMG1, SpHMG1, ERG10, ERG12 (YlMVK), ERG8 (YlPMK), YlMVD1, ERG20 (YlFPP), YlPOT1 and YlPAT1 genes were obtained by PCR reaction using specific optimized-primer by Integrated DNA Technologies Company, USA (Supplementary Table S3). AaADS was amplified by primer pair AaADS_UP F and AaADS_Dwn R. All used genes contained the intron that removed for designing primer and amplify genes. The PCR product was introduced at the *SnaBI* and *KpnI* digested site of pYLXP’ and using Gibson Assembly method. The start codon of each gene was substituted by a “TAACCGCAG” sequence to complete the intron fragment (Wong et al., 2017).

The YaliBrick standards was used for assembly amorphadiene pathway into pYLXP’ derived plasmids (Wong et al., 2017). Generally, for digestion and introducing the genes in each structure *ClaI*/*AvrII* were used for recipient plasmids while *ClaI*/*NheI* used to treatment of the donor plasmids*.* And T4 ligation was used to prepare monocistronic cassettes. *E. coli* NEB5α transformation and mostly *KpnI/XhoI* digestion were used for screening the desired cassettes. All plasmids included in this research were shown in Supplementary Table S4*.*

### 4.3 *Y. lipolytica* transformation

*Y. lipolytica* transformation was used based on lithium acetate (LiAc) method. Frozen *Y. lipolytica* Po1g was streaked on YPD plate and grown at 30 ​°C for at least 16 ​h. The yeast transformation mixture was prepared using 90 ​μL 50% PEG4000, 5 ​μL 2 ​M LiAc, 5 ​μL single strand DNA (salmon sperm DNA solution), and 200–500 ​ng plasmid DNA. For high transformation efficiency, single-strand DNA should be boiled and cooled down before adding to the transformation buffer. The solution mixture should be mixed by vortex for at least 15 ​s before use. The yeast cells were scraped and mixed to the solution buffer and completely mixed by vortexing for at least 15 ​s. Vortexing for 15 ​s every 10 ​min, the master transformation buffer was incubated at 30 ​°C for 30–45 ​min and subsequently at 39 ​°C for 10 ​min to boost transformation performance. The transformation mixture should vortex for 15 ​s every 10 ​min ​at each step. Finally, the transformed cells were plated on selective plate (CSM-Leu) and incubated at 30 ​°C for 2–3 days.

### 4.4 Growth condition and fermentation cultivation

For pre-culture single colonies were inoculated from fresh plates in 3 ​mL regular drop-out mix synthetic minus leucine culture media and grown for 48 ​h ​at 30 ​°C, 250 ​rpm agitation and 5 ​cm orbit cast. To carry out fermentation in 250 ​mL shaking flask, the appropriate amount of pre-culture was inoculated to 40 ​mL nitrogen-limited complete synthetic media (C/N ​= ​80) and keep at 30 ​°C for 144-h, 220 ​rpm. After 48 ​h 8 ​mL (20% v/v) dodecane was injected to each shake flasks to trap amorphadiene. To analyze the effect of cerulenin a final concentration of 1 ​mg/L cerulenin was added at 48 ​h.

### 4.5 Analytical method

Samples were taken every 24 ​h starting at 72–144 ​h. 500 ​μL of upper phase was transferred into 1.5 ​mL microcentrifuge tube. 2.5 ​μL β-caryophyllene of 10 ​mg/ml stock was added to each tube. Each sample was vortexed for approximately 1 ​min and then centrifuged at 14,000 ​g for 5 ​min. 200 ​μL from the upper layer transferred into 2 ​mL ​GC vials. A gas chromatography–flame ionization (GC-FID) was operated in an Agilent 7820A. HP-5 column (30 ​m ​× ​320 ​μm ​× ​0.25 ​μm) was used for separation of compounds, using helium as the carrier gas with a linear velocity of 2 ​ml/min. The column temperature profile was 100 ​°C for 2 ​min, 25 ​°C/min increase to 200 ​°C, 20 ​°C/min increase to 280 ​°C and 280 ​°C for 2 ​min. Amorphadiene production was characterized by comparison with β-caryophyllene as a trustworthy internal standard. The GD-FID results according compare with two standards confirmed retention time at 5.5 and 5.6 ​min are for presence of β-caryophyllene and amorphadaiene, respectively.

## Author contributions

PX conceived the topic and designed the study. MM performed genetic engineering and fermentation experiments. MM wrote the manuscript. PX revised the manuscript.

## Declaration of competing interest

A provisional patent has been filed based on the results of this study.
