# Supplementary material for: Debottlenecking mevalonate pathway for antimalarial drug precursor amorphadiene biosynthesis in Yarrowia lipolytica
Source: Metab Eng Commun. 2020 Jan 2;10:e00121. doi: 10.1016/j.mec.2019.e00121 (PMC6957783; doi:10.1016/j.mec.2019.e00121)
Supplement: Multimedia component 1 [file mmc1.pdf]

Debottlenecking mevalonate pathway for antimalarial drug precursor amorphadiene biosynthesis in *Yarrowia lipolytica*

Monireh Marsafari, Peng Xu*

Department of Chemical, Biochemical and Environmental Engineering, University of Maryland Baltimore County, Baltimore, MD 21250

* Corresponding author Tel: +1(410)-455-2474; fax: +1(410)-455-1049. E-mail address: pengxu@umbc.edu (PX)

Table S1. Genes used in this paper.

Gene	Origin	Database and accession number	Optimization	References
AaADS	*Artemisia annua*	NCBI: AEQ63683.1	Yes	
YlHMG1	*Yarrowia lipolitica* Po1g	UniProtKB: Q6C704	Yes	(Huang et al., 2018)
tYlHMG1	*Yarrowia lipolitica* Po1g	UniProtKB: Q6C704	Yes	
SpHMG1	Streptococcus pneumoniae	UniProtKB - Q8DNS5	Yes	
ScHMG1	*Saccharomyces cerviciae*		Yes	(Basson, Thorsness, & Rine, 1986)
ERG13	*Yarrowia lipolitica* Po1g	GRYC[1]: YALI0F30481g	Yes	(Martin, Pitera, Withers, Newman, & Keasling, 2003)
ERG8	*Yarrowia lipolitica* Po1g	GRYC: YALI0E06193g	Yes	(Martin et al., 2003)
ERG10	*Yarrowia lipolitica* Po1g	GRYC: YALI0B08536g	Yes	(Paddon et al., 2013)
ERG12	*Yarrowia lipolitica* Po1g	GRYC: YALI0B16038g	Yes	(Martin et al., 2003)
MVD1	*Yarrowia lipolitica* Po1g	GRYC: YALI0F05632g	Yes	(Martin et al., 2003)
ERG20	*Yarrowia lipolitica* Po1g	GRYC: YALI0E05753g	Yes	(Westfall et al., 2012)
POT1	*Yarrowia lipolitica* Po1g	GRYC: YALI0E18568g	Yes	
PAT1	*Yarrowia lipolitica* Po1g	GRYC: YALI0E11099g	Yes	

(1) GRYC refers to the genome resources for yeast chromosomes (http://gryc.inra.fr/index.php?page=home).

Table S2. Strains constructed in this paper.

Strain	Annotation	References
Po1g/ADS	Po1g containing pYLXP'-AaADS, used for the optimization of amorphadiene production	This work
Po1g/AYlH	Po1g containing pYLXP'-AaADS-YlHMG1, used for the optimization of amorphadiene production	This work
Po1g/AtYlH	Po1g containing pYLXP'-AaADS-tYlHMG1, used for the optimization of amorphadiene production	This work
Po1g/ASpH	Po1g containing pYLXP'-AaADS-SpHMG1, used for the optimization of amorphadiene production	This work
Po1g/AScH	Po1g containing pYLXP'-AaADS-tScHMG1, used for the optimization of amorphadiene production	This work
Po1g/AYlHERG8	Po1g containing pYLXP'- AaADS-YlHMG1-ERG8, used for the optimization of mevalonate pathway	This work
Po1g/AYlHERG12	Po1g containing pYLXP'- AaADS-YlHMG1-ERG12, used for the optimization of mevalonate pathway	This work
Po1g/AYlHMVD1	Po1g containing pYLXP'- AaADS-YlHMG1-MVD1, used for the optimization of mevalonate pathway	This work
Po1g/AYlHERG20	Po1g containing pYLXP'- AaADS-YlHMG1-ERG20, used for the optimization of mevalonate pathway	This work
Po1g/AYlH1$_{x2}$E13E10E12	Po1g containing pYLXP'- AaADS-YlHMG1$_{x2}$- ERG13-ERG10-ERG12 used for the optimization of amorphadiene production	This work
Po1g/AYlH1$_{x2}$E13E10E12E8	Po1g containing pYLXP'- AaADS-YlHMG1$_{x2}$- ERG13-ERG10-ERG12-ERG8 used for the optimization of amorphadiene production	This work
Po1g/A$_{x2}$tYlH	Po1g containing pYLXP'- AaADS$_{x2}$-tYlHMG1 used for the optimization of amorphadiene production	This work
Po1g/A$_{x2}$tYlHE12	Po1g containing pYLXP'- AaADS$_{x2}$-tYlHMG1-ERG12 used for the optimization of amorphadiene production	This work
Po1g/A$_{x2}$tYlHP	Po1g containing pYLXP'- AaADS$_{x2}$-tYlHMG1-YlPOT1 used for the optimization of amorphadiene production	This work
Po1g/A$_{x2}$tYlHE12P	Po1g containing pYLXP'- AaADS$_{x2}$-tYlHMG1-ERG12-YlPOT1 used for the optimization of amorphadiene production	This work
Po1g/A$_{x2}$tYlHPP	Po1g containing pYLXP'- AaADS$_{x2}$-tYlHMG1-YlPOT1-YlPAT1 used for the optimization of amorphadiene	This work

	production	
Po1g/A$_{x2}$tylHE12PP	Po1g containing pYLXP'- AaADS$_{x2}$-tYlHMG1-ERG12-YlPOT1-YlPAT1 used for the optimization of amorphadiene production	This work

Table S3. Primers used in this paper.

Primer	Sequence (5’-3’)
YlHMG1 F	GCACTTTTTGCAGTACTAACCGCAGCTACAAGCAGCTATTGGAAAGATTGTGG
YlHMG1 R	GGCCATGGAACTAGTCGGTACCCTATGACCGTATGCAAATATTCGAACC
tYlHMG1 F	GCACTTTTTGCAGTACTAACCGCAGCTACGAGAAGTTGTGCGAACCCAGTCTG
tYlHMG1 R	GCCATGGAACTAGTCGGTACCCTATGACCGTATGCAAATATTCGAACC
SpHMG1 F	CCAGCACTTTTTGCAGTACTAACCGCAGACAGGGAAAACCGGCCATATAG
SpHMG1 R	ACAGGCCATGGAACTAGTCGGTACCTCATGTATTTTCCAGAACCTGCTTC
tScHMG1 F	AGCACTTTTTGCAGTACTAACCGCAGGACCAGCTTGTTAAAACAGAAGTA
tScHMG1 R	GACAGGCCATGGAACTAGTCGGTACCTTATGATTTTATGCAGGTTACTGA
ERG13 F	CCGACCAGCACTTTTTGCAGTACTAACCGCAGTCGCAACCCCAGAACGTTGG
ERG13 R	GGACAGGCCATGGAACTAGTCGGTACCGATGACGAACTAGAGCATGAAAG
ERG10 F	CCGACCAGCACTTTTTGCAGTACTAACCGCAGGAGCCCGTCTACATTGTTTCTAC
ERG10 R	GGACAGGCCATGGAACTAGTCGGTACCCTAACACTTCTCAACAATGATAGA
ERG8 F	CCGACCAGCACTTTTTGCAGTACTAACCGCAGACCACCTATTCGGCTCCGGGAAAG
ERG8 R	GGACAGGCCATGGAACTAGTCGGTACCCTACTTGAACCCCTTCTCGAGCCG
ERG12 F	CCGACCAGCACTTTTTGCAGTACTAACCGCAGGACTACATCATTTCGGCGCCGAG
ERG12 R	GGGACAGGCCATGGAACTAGTCGGTACCCTAATGGGTCCAGGGACCGATG
MVD1 F	CCGACCAGCACTTTTTGCAGTACTAACCGCAGATCCACCAGGCCTCCACCACCGC
MVD1 R	GGACAGGCCATGGAACTAGTCGGTACCCTACTTGCTGTTCTTCAGAGAACC
ERG20 F	CCAGCACTTTTTGCAGTACTAACCGCAGGCTTCAGAAAAAGAAATTAGGAGAG
ERG20 R	GGACAGGCCATGGAACTAGTCGGTACCCTATTTGCTTCTCTTGTAAAC
YlPOT1 F	CCGACCAGCACTTTTTGCAGTACTAACCGCAGGACCGACTTAACAACCTCGCC
YlPOT1 R	GGACAGGCCATGGAACTAGTCGGTACTTACTCGGCAACAACCAGAGAAGC
YlPAT1 F	GACCAGCACTTTTTGCAGTACTAACCGCAGCGACTCACTCTGCCCCGACTTAAC
YlPAT1 R	GGACAGGCCATGGAACTAGTCGGTACCTACTCGACAGAAGAGACCTTCTTG

Table S4. Plasmids used in this paper.

Plasmid	Annotation
pYLXP’	YaliBrick plasmid, used for pathway assemble
pYLXP’-AaADS	For the construction and optimization of amorphadiene production
pYLXP’-AaADS-YlHMG1	For the construction and optimization of amorphadiene production
pYLXP’-AaADS-tYlHMG1	For the construction and optimization of amorphadiene production
pYLXP’-AaADS-SpHMG1	For the construction and optimization of amorphadiene production
pYLXP’-AaADS-tScHMG1	For the construction and optimization of amorphadiene production
pYLXP’- AaADS-YlHMG1-ERG8	For the construction and optimization of mevalonate pathway
pYLXP’- AaADS-YlHMG1-ERG12	For the construction and optimization of mevalonate pathway
pYLXP’- AaADS-YlHMG1-MVD1	For the construction and optimization of mevalonate pathway
pYLXP’- AaADS-YlHMG1-ERG20	For the construction and optimization of mevalonate pathway
pYLXP’- AaADS-YlHMG1$_{x2}$-ERG13-ERG10-ERG12	For the construction and optimization of mevalonate pathway and amorphadiene production
pYLXP’- AaADS-YlHMG1$_{x2}$-ERG13-ERG10-ERG12-ERG8	For the construction and optimization of mevalonate pathway and amorphadiene production
pYLXP’- AaADS$_{x2}$-tylHMG1[1]	For the construction and optimization of amorphadiene production
pYLXP’- AaADS$_{x2}$-tylHMG1-ERG12	For the construction and optimization of amorphadiene production
pYLXP’- AaADS$_{x2}$-tylHMG1-ylPOT1	For enhancing acetyl-CoA synthesis and improvement of amorphadiene production
pYLXP’- AaADS$_{x2}$-tylHMG1-ERG12-ylPOT1	For enhancing acetyl-CoA synthesis and improvement of amorphadiene production
pYLXP’- AaADS$_{x2}$-tylHMG1-ylPOT1-ylPAT1	For enhancing acetyl-CoA synthesis and improvement of amorphadiene production
pYLXP’- AaADS$_{x2}$-tylHMG1-	For enhancing acetyl-CoA synthesis and improvement

ERG12- ylPOT1-ylPAT1	of amorphadiene production

(1) The subscripts "$_{x2}$" refer to gene copy number in the plasmids.

Supplementary figures

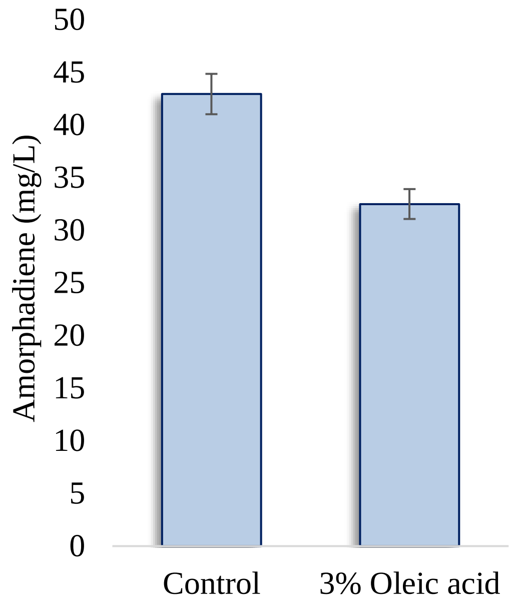

Supplementary Figure S1. Effect of 3% oleic acid on amorphadiene production carried out for Po1g/AYlH. CSM-Leu media without Oleic acid used as a control condition

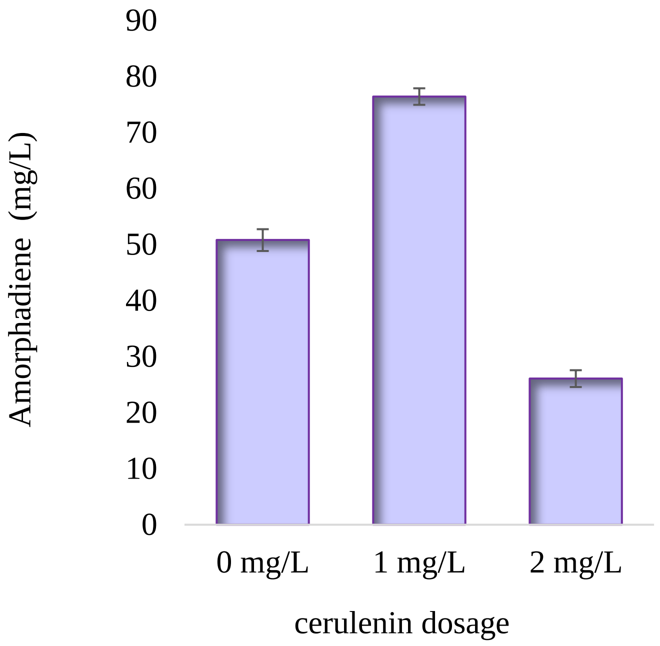

Supplementary Figure S2. Cerulenin optimization dosage carried out for Po1g/AYlH strain in leucine drop-out complete synthetic media (CSM-Leu).

References

Basson, M. E., Thorsness, M., & Rine, J. (1986). Saccharomyces cerevisiae contains two functional genes encoding 3-hydroxy-3-methylglutaryl-coenzyme A reductase. *Proc Natl Acad Sci U S A, 83*(15), 5563-5567.

Huang, Y. Y., Jian, X. X., Lv, Y. B., Nian, K. Q., Gao, Q., Chen, J., . . . Hua, Q. (2018). Enhanced squalene biosynthesis in Yarrowia lipolytica based on metabolically engineered acetyl-CoA metabolism. *J Biotechnol, 281*, 106-114. doi:10.1016/j.jbiotec.2018.07.001

Markham, K. A., Palmer, C. M., Chwatko, M., Wagner, J. M., Murray, C., Vazquez, S., . . . Alper, H. S. (2018). Rewiring *Yarrowia lipolytica* toward triacetic acid lactone for materials generation. *Proceedings of the National Academy of Sciences of the United States of America, 115*(9), 2096-2101. doi:10.1073/pnas.1721203115

Martin, V. J., Pitera, D. J., Withers, S. T., Newman, J. D., & Keasling, J. D. (2003). Engineering a mevalonate pathway in Escherichia coli for production of terpenoids. *Nat Biotechnol, 21*(7), 796-802. doi:10.1038/nbt833

Paddon, C. J., Westfall, P. J., Pitera, D. J., Benjamin, K., Fisher, K., McPhee, D., . . . Newman, J. D. (2013). High-level semi-synthetic production of the potent antimalarial artemisinin. *Nature, 496*(7446), 528-532. doi:10.1038/nature12051

Westfall, P. J., Pitera, D. J., Lenihan, J. R., Eng, D., Woolard, F. X., Regentin, R., . . . Paddon, C. J. (2012). Production of amorphadiene in yeast, and its conversion to dihydroartemisinic acid, precursor to the antimalarial agent artemisinin. *Proc Natl Acad Sci U S A, 109*(3), E111-118. doi:10.1073/pnas.1110740109

Zha, W. J., Rubin-Pitel, S. B., Shao, Z. Y., & Zhao, H. M. (2009). Improving cellular malonyl-CoA level in *Escherichia coli* via metabolic engineering. *Metabolic Engineering, 11*(3), 192-198. doi:10.1016/j.ymben.2009.01.005
